# Address, Delivered before the Society of the Alumni of the Baltimore College of Dental Surgery

**Published:** 1849-04

**Authors:** J. H. Foster

**Affiliations:** New York.


THE AMERICAN

### Journal of Dental Science

Vol. IX.] APRIL, 1849. [No. 3.

### ARTICLE I.

*Address, delivered before the Society of the Alumni of the Baltimore College of Dental Surgery, at their First Annual Meeting.* By J. H. Foster, M. D., of New York.

——"our places,
The way of our profession, is against it;
We are to cure such sorrows, not to sow them."
*King Henry VIII.*

*Mr. President and Gentlemen:*

True science is of slow growth. It is our privilege to live in an age of progressive and rapid improvement. There is a continually increasing motive power which propels the mind of man to seek out and develop all the great agents in the laboratory of nature that an all-wise Providence purposed for his use and advancement.

It seems, truly, as if all things were indeed to be brought in subjection, and made subservient to the will of man: that nothing upon the face of the earth was to remain undiscovered, or lie in its bosom undeveloped. It is, in truth, *the Golden Age;* the march of mind is onward, and as it must, and ever will be, to the greatest extent, "The march of mind is with the free."

Do we realise at the present day the advancement in man's progressive destiny? So many strange and important discoveries have been brought to light, that we have almost ceased to wonder at any new revelation.

It is now near the middle of the nineteenth century, and within comparatively how recent a period have many of the great discoveries, the vast improvements in the arts and sciences, been made, by which unexplored wildernesses have been penetrated, mountains unlocked, the desert places cultivated; by which rivers have been threaded to their sources; by which the vast expanse of ocean has been traversed, bearing the strong hearts and adventurous spirits of the civilized and the free, carrying light and knowledge, to the neglected shores of the heathen and the barbarian.

By the power of steam, and the facility of communication which it has afforded, he has broken down the barriers of time and space, and been enabled to explore all the recesses of the world, and illuminate all its dark corners.

By the invention of the magnetic telegraph, places remote from each other are brought in close proximity, so that man, from his home on the shores of the Atlantic, may soon hold converse with his fellow-man on the borders of the far Pacific. The press, rail roads, and electro-magnetic wires, are great agents for the spread of intellectual light. Wherever these extend, the darkness of ignorance is rapidly dispelled, and the light of knowledge irradiates the land.

Our advance as a nation, in the improvements of the age, has been unparalleled in the history of the world, and it has been owing more to the spread of knowledge mong the people, than to any other cause. There is that spirit of freedom to think and to act, possessed by every individual, whatever may be his occupation, which, if his pride and affection are merged in it, will impel him to continual exertion to improve himself and honor his calling, and, by new inventions and discoveries, increase his knowledge and usefulness.

A man may boast that he obeys his country's laws, may acknowledge it a duty to act whenever called upon to aid in their administration, and in defending her against foreign invasion; for this he may be considered a citizen, and entitled to all the rights of citizenship; but there is one other important condition, which should be understood and complied with, without which he is not a good citizen in any community—viz. *occupation*, some employment by which he can be useful to his fellow-men, and honor himself.

He who is not disposed to perform some duty, to do some work, in this active, working-day world of ours, is unconscious of how great a privilege and source of happiness he deprives himself. There are those in every community, having the natural talents and abilities actively to engage in some undertaking, for the improvement and elevation of our race, who waste their lives, neglecting and abusing their privileges. Those only who join serious occupation with pleasure, enjoy life as they should. Mere pleasure cannot be the business of a man of sense and character. The devotee to pleasure and ennui is as much the slave to false and erroneous impressions of the true source from whence pure happiness is to be derived, as is the slave of intemperance, whose whole soul is centered in self-gratification, imbibed from the intoxicating bowl.

Self-indulgence is the main-spring of their inclinations. Their connections, their relations to society, have but a frail attachment upon their affections, and reasoning from analogy, if they have love for none of these, they can have but little for their country. He cannot feel that he has a country, who is doing nothing by his own exertions to advance it. He is a foreign substance, a dead weight upon the body politic. If all were to act thus, what would become of our laws, our enterprise, our national improvement, not to say existence?

> "Lives there the man with soul so dead,
> Who never to himself hath said,
> This is my own, my native land!"

Those who pass through life thus inactive, even though it were to them a never-ending pageant, though it were their lot to dwell in marble palaces, "where nought but what is costly flourishes," amid affluence and luxury, surrounded with rank and wealth, possessed of everything which can gratify the taste or minister to the appetite, know not and can never realise the increased happiness there is in a life of useful, well-directed, active cultivation of the faculties and energies of man's nobler nature. They can never be impressed with this truth: that perseverance, and a fixed determination to devote to the final accomplishment of a laudable object, those energies and talents with which we are gifted for wise and useful ends, at all times reward us for our exertions, in the deep satisfaction we ourselves feel, and in the approval of our fellow-men.

There is no mechanical occupation, be it ever so humble, that may not be made to confer honor upon a man, and the occupation itself become ennobled, if men of industry, skill, enterprise, honesty, perseverance, determination, are engaged in it

We need no stronger evidence of this, than is furnished in the history of our own calling. In no other professional or mechanical pursuit, has there been greater improvement, or a more rapid increase of well-instructed and skilful workmen.

Within how short a time, how incomparably transient a period, has this great change been wrought. I need not ask you to contrast its present position with its former, shall I say degraded, condition? But a few years since, and how were its followers viewed? how treated? To a great extent, as artisans engaged in an occupation of such a nature as to require the utmost secrecy. They were only to be recognized in their workshops, as if it were something disgraceful, humiliating to employ them. To what was this owing? In part to a morbid and depraved mawkish sentimentality, but more to the illiterate, unrefined, uneducated, unprincipled character of a majority of those who professed to be practitioners of dental surgery. "*Tempora mutantur ab illa.*"

Now I ask, gentlemen, how has this great and favorable change been brought about, under such untoward circumstances, and with such manifestly powerful opposing obstacles to the march of improvement?

How is it that it has become a great, a useful, a scientific body, so that its members proper obtain the confidence, the gratitude, the respect, intercourse, association and communion with all classes, and all ranks in society? That it is no longer a mechanical employment merely, but that it has become an honorable professional calling?

It was because the tree of knowledge had been planted that we and our posterity might gather its fruits. To a few liberal minded men, ardent in the pursuit of all that science and art could furnish to enrich their labors, and establish for them a character as philanthropists and benefactors of their race, we owe much of our success.

May we always remember with gratitude our obligations to them, for the labor, the zeal, the energy which they displayed. The fidelity with which they discharged their trust, their untiring devotion in the path of duty, for the attainment of a more lofty, elevated and honorable position, upon which to raise this structure of their hopes, is worthy of our contemplation. Who does not experience the good effects of their efforts? Who does not realise the advantages of the change which is so apparent? Who does not rejoice in beholding the annual increase of well-instructed members of the profession, and the corresponding protection thereby afforded to the community, in the security and safety of our operations? Who does not feel that the reformation which is progressing in the character, fitness and capacity of our practitioners, will be continual, and must inevitably tend to the increased honor and respectability of the profession?

There is an *esprit de corps*, a sense of professional pride, which, to a greater or less extent, must animate the spirit of every high-minded man, who is ambitious of distinction, who is aiming to be great among us, which is commendable. It should be cultivated.

It is this which excites our admiration for the character of the founders of our art, which impels us to a veneration of their works and virtues, and induces us to cherish their memories.

I have ever looked with admiration and a feeling of pride, upon the works of those who have gone before us, as well as of those whose names stand deservedly high upon the scroll of fame, who are still among us; but I knew not the extent to which the feeling I have spoken of pervaded my own breast, and exerted its influence, until a few weeks since.

I was somewhat suddenly and unexpectedly introduced to a gentleman of our profession from Europe. He is travelling in this country for improvement, as well as pleasure, and I am gratified to recognize him among my audience here to day.

I said my introduction was sudden, and at the mention of his name, I felt, as suddenly, an increased action in my veins, and my heart's blood grow warmer within me, to take by the hand, the son of a gentleman so eminently, and deservedly, distinguished, as Dr. Robert Nasmyth of Edinburgh, Scotland.

There are occasions, gentlemen, when it is proper, when it is profitable, to pause in the career of life, not only to mark the progress of events, but to observe the character of men distinguished for eminent success or signal failure, that we may emulate the examples of the one, and shun the misfortunes of the other.

We can account with some degree of certainty for the success of the few who have attained high rank and station; we know that it has cost them years of unremitting toil, and that it has been by untiring application, long continued attention, and a singular love of labor, that they now stand, as splendid examples, of what genius and industry may accomplish.

On the other hand, we can, with equal certainty, account for the ill success of thousands, who have not attained even mediocrity, and a large proportion of whom are a disgrace to the profession.

How common a thing it is to hear remarks respecting the *uncertainty* of dental operations. Individuals who have been so unfortunate as to have passed through the hands of one or more practitioners of the latter class, without having derived the expected benefit, having nearly lost all confidence in the availability of the art, are very reluctant to believe, that there is any certain permanent utility in it, or that there can be any difference among operators, in point of education and skill.

There are others, peradventure, who seem to think that dental operations have been brought to a state of perfection, who, having always been fortunate in receiving aid from the skilful, express the greatest astonishment, if the operator in whom they have placed confidence, fails of success in one instance.

Our art is not yet omnipotent. The most expert and successful practitioners must sometimes fail, and that, too, in cases which do not require any great degree of extraordinary skill. Perhaps oftener in these, than in operations of a more difficult and complex character. They are never surprised and confounded, when some wonder stricken patient rushes in, with a countenance and manner evincing the utmost consternation and astonishment, (and as sometimes happens, inconsiderately, in the presence of others,) exclaims, *Doctor, one of your fillings has come out!*

As well might the skilful physician, who fails, as all must, here and there, in an extensive practice, to restore to health, a few of his patients, be held accountable for his want of success in all cases. The knife of the surgeon does not always bring the life current into healthy action, and restore the body to its pristine vigor, when it lops off a diseased member.

There is *a degree of uncertainty* in medicine, and in surgery, in all its branches. They claim, in medical diction, "to be the application of such means, as accumulated experience, cautious observation, and sound judgment, have proved efficacious in ameliorating, or curing disease; and their perfection would consist, in a knowledge of the most certain, ready, and least painful mode of restoring health, when restoration is possible, and of the means of soothing pain, and prolonging life to the utmost, when restoration is impossible."

To the most skilful of our profession, uncertainty, in a large majority of their operations, is a word that cannot be applied—our art may yet approximate closely to perfection, and even now there can be no question that uncertainty is more in the *practitioner*, than in the practice itself.

*Why* are there so few competent practitioners among so many pretenders in this science?

One great cause is, the imperfection of existing knowledge. Young men enter upon the pursuit of a knowledge of dental surgery, with the impression, too often, that it is the easiest thing in the world to acquire, that one is as competent as another to give instruction. They have no adequate conception of its importance. Unconscious that intensity of application, and long continued study are requisite before they can succeed in acquiring, with the best instructors, correct principles; and that by years of patient industry, and persevering assiduity, they can alone expect to put those principles into successful practice. Another cause is,

*Constitutional unfitness* and *incapacity* to acquire such knowledge. There are, comparatively, few applicants who could ever become first class operators and of these many acting upon erroneous impressions at the outset, deceived by those who profess to be qualified to give instruction, seek to gain at a cheap rate, and in far too short a time, knowledge which others with better opportunities, find it hard to acquire by years of application.

It is, comparatively, an easy thing for a student to acquire such knowledge of principles as does exist; ability to reduce to practice that knowledge is a very different, and a very difficult thing.

It is conceded, that the operation of stopping, plugging, or filling teeth, (I wish the profession would unite upon some one of these words, to express the meaning of the phrase,) is by far the most difficult operation, to learn to do well, and there are very few, who, with the best advantages, succeed in becoming expert and skilful.

Every capable experienced practitioner knows, that there is no goal, no limit to improvement in this. I will venture to say, there is not one, who, taking a retrospective glance, but can look back from time to time with joyful satisfaction at his improvement; although at the various periods, from which he may draw his data, he had indulged in vain imaginings, that there was for him but little more room for improvement, that he had nearly attained perfection; and then wonder himself, how he could have been successful, when careless in this or that little principle of practice, which he now deems to be of such essential importance.

He who feels that there is no more room for improvement will be likely to retrograde in his calling.

Before correctness, facility and success, can be attained in any profession or employment, the mind and its instruments must be well trained to act harmoniously, and continuously in its pursuit.

There have been, doubtless, many who have commenced practice with limited knowledge, hoping and expecting to acquire correct principles by experience, honestly intending to do right, as far as they knew how, in alleviating human suffering.

Few, *very few*, have been successful by their own unaided exertions, and active energies, in attaining this end. These are entitled to, and receive the greater commendation, that they have overcome all obstacles, and triumphed under such disadvantages. It is, nevertheless, unfortunate for the community, that a vast majority of those who make the attempt, did not realize, how hopeless an undertaking they had engaged in, and how much better fitted they were, by nature and education, for some other employment.

There are, on the other hand, many ignorant pretenders, sensible of their deficiency in knowledge and capacity, wilfully bent upon imposition, caring not for the success of their operations, if they can only succeed in obtaining their fee, studious only to acquire new patients, never expecting long to retain old ones.

Cases of gross imposition, and mal-practice combined, every respectable practitioner has almost daily cognizance of; and yet, even this class of operators are often enabled to impress a community with a belief in their superior qualifications. It is thus they multiply and expand, and for a brief period, will sometimes eclipse and outshine, the modest, unpretending man of merit, so that,

"Vice triumphs, virtue falls,
Justice is charmed by error's song."

"But evil hath a bound,
And truth o'erthrown itself shall right;
Vice oft by sightless men is crowned:
But lo! it withers in its might,
And creeps confounded, in the dazzling light."

There is yet one other class of operators, whose opportunities, whose means of degrading the profession are more ample and more fatal.

The well instructed, well educated, enlightened, yet unprincipled; men who are capable and scientific, have attained high rank and station, and who having gained the confidence of the community, act in direct violation of right, using impure materials, where the purest only should be used, and that sometimes surreptitiously. These are mal-practitioners of the worst description, in asmuch as they exert a powerful influence in increasing, and encouraging, that class of operators last mentioned.

There may be among those who use the *mineral amalgam* some who honestly believe in its utility, but I cannot conceive how it is possible for any one of superior skill and judgment to continue such a practice long. Leaving the question, however, as to their conscientiousness, there are many more who use it, professing to do so in extreme cases only, who substitute it for gold, where they know, gold is the only material that should be used.

As to tin, and other malleable metals, although I have never approved of any thing but gold foil, and have never seen the necessity of using any thing else, I should not object so strongly to these, as to the other compound, if they were always used with the *knowledge* and *consent* of the patient. But they are not; they are sometimes inserted secretly into cavities, and the wrong concealed by a covering of gold. Of all modes of practice this is the most criminal and detestable. Men who can be guilty of this act, have no regard for their own character, or that of the profession. Does conscience become inactive? What is the object? What the gain? For what great stake do they play this small game? To save a little gold? Aye! sacrifice honor, reputation, a good name! "For as much trash as may be grasped in the hand, thus."

Having spoken of the defective nature of dental education, and practice, I pass to a consideration of some of the causes which retard its reformation. The success which attends malpractice is attributable, in no small degree, to the direct influence of men of high standing in society, who, through a friendly intimacy, or acquaintance, lend their names without knowing whether this act of intentional kindness to one individual, may not prove a serious injury to thousands.

How often do we see the names of magistrates, doctors of divinity, doctors of laws, and even doctors of medicine, from whom the community have a right to expect the greatest care and circumspection, attached to *certificates of recommendation.*

I regret to make the assertion, but I have noticed, as far as my observation has extended, that regular practitioners of medicine, too often, pay but little attention to diseases of the teeth, and their direct, or indirect sympathetic connexion with, or influence upon, other organs.

Cases do occur, in which an examination of the *teeth* instead of the *tongue,* might enable physicians to form a more correct prognosis of disease, and its causes, and be the means of saving their patients from repeated, and protracted sickness and suffering.

Here is a wide field of investigation open to the inquiring mind. We have hardly begun to estimate the extent to which the teeth affect the human system.

The stomach which more directly sympathises with an unnatural, or unhealthy condition of the teeth, suffers more immediately, and through this, other organs, more remote, become involved. I have heard a lady say, "that she had not eaten so well, slept so well, felt so well, or enjoyed life so much for thirty years, as she had done since she had lost her imperfect natural teeth, and had an artificial set substituted."

Another lady, within a few weeks, remarked, that she had experienced a great change in her health, during the five months, she had been using an entire set of artificial teeth; that during the preceding years she had suffered, every few weeks, from attacks of dyspepsia, nervous head-ache, &c. and was continually sending for her physician, and taking medicine to obtain relief.

She wished to know, if the change was attributable to her being able so much better to masticate her food?

Who has not had in his experience, examples of such cases?

There is one distinguished member of the profession present, who could relate to you the particulars of a case, which occurred many years since, of a lady, whose life he was instrumental in saving by the extraction of several diseased teeth. It was a confirmed case of consumption; extension of life, beyond the space of a few short months, was considered hopeless. She began to improve from that very day, and her physician, an eminent practitioner, in that section of the country, as well as her family and friends, attributed her recovery to the performance of this operation; submitted to, in her weak and feeble condition, that the tranquillity of her latter days might be uninterrupted, and her peace of mind secured, by freedom from bodily pain and suffering.*

At the present day, most respectable physicians are impressed with the importance of this branch of the healing art. They feel it to be a duty, to know something respecting the nature and tendency of our operations, that they may be the better able to judge of the comparative merits of different operators.

How many people there are who make it a point to consult their physician, and who depend very much upon his advice, and judgment, in selecting a dental operator.

Every physician assumes a weighty responsibility in advising upon this question, and no physician, priest, or layman, has a moral right to lend his name to any one whose operations he has not himself tested, unless by other good and sufficient evidence, he has been convinced of their security and safety.

The press has been, and is, a powerful agent for the spread of malpractice.

Every publisher, and editor, is at liberty to insert all advertisements sent to his paper, it is his legitimate occupation, and one of the principal sources of emolument; but he has not a moral right to certify or endorse

* This gentleman has since reminded me of a similar case, which occurred in his own family, under like circumstances, and with the same happy result.

the professions made by self-idolized pretenders, as *truth*.

Yet how often do we read advertisements of the most gross and infamous description, (intended to impose upon the credulity of that portion of the community, who are always eager for information, gained from this source,) strengthened and supported by newspaper puffs, and editorial commendation, when, if the truth were known, the editor knows no more of the pretender's merits, than he has gleaned from the advertisement itself.

While we admit that through the medium of the public press, a mass of useful information is continually furnished to every class of our citizens, and rejoice that it is so, it unfortunately happens, that it is also a medium through which quackery of every description is introduced.

But the mass of the people are increasing in wisdom and knowledge, experience has taught many, how little dependence can be placed in *advertisements*, from our profession; and unless we are greatly deceived, the signs of the times will justify the prediction, that the period is not far distant, when respectable men will not be found willing agents, in the work of imposition and fraud.

There is a portion of mankind, who have neither leisure nor inclination to reflect or reason, credulity is more convenient to them, than the researches necessary for the investigation of truth, and so long as they will be imposed upon, there will never be wanting those who will contrive the ways and means. Go where you may, throughout the country, and the footsteps of the destroyer may be traced.

He levies his tax upon all classes, from the judge upon his bench, to the peasant who earns his bread by the sweat of his brow.

In some of the governments of Europe, charlatanism is restrained by laws which subject the offender to severe penalties; but in a government like ours, where the people make and administer their own laws, a statute which does not harmonise with public sentiment, will be repealed, or not enforced.

The public mind must be gradually enlightened, to become impressed with the importance of the subject.

Let the community be made to realise, to the full extent, the evil, and the ruinous consequences attendant upon the operations of a large proportion of practitioners, and in due time laws may be enacted, and restrictions imposed, which will have the desired effect.

*Societies* and *associations* have been formed for the avowed purpose of improving and elevating the profession—of controlling and restraining these evils.

Without any knowledge, Mr. President, of the preliminary steps to the formation of this association, or its intended course of action—uninformed what system your individual or collected opinions may have adjudged as the most likely means to promote the best interests of the community—I shall plainly and explicitly present my own opinions as to the utility of associations in general, and comment upon the prominent features of one or two in particular, which deserve some notice on an occasion like this. I present my views to your consideration, with all due deference to the superior judgment of those who are older and more experienced than myself. If a single idea is suggested which may not have occurred to you in your deliberations, and it be deemed sufficiently important to influence your future action, I shall be amply compensated, if in the smallest degree I have aided in the praiseworthy object of your association.

The inquiry naturally arises, What good has been effected heretofore, by *societies*, in our profession? How have they been organised? conducted? and how have they succeeded? and of those which still exist, what is their present position?

The history of the past furnishes but a melancholy and discouraging picture of experiments. Many local societies have been formed, which, after struggling through a brief existence, have expired; and I believe there are but three of any extended influence, now living, to be considered.

This is a very unsatisfactory inducement to the formation of a new association; unless we see and avoid the shoals upon which others have been stranded.

To what cause shall we attribute these failures? I answer, *chiefly* to the want of sufficient material of a proper character to form a solid fabric; and to the introduction, as a substitute, of such as could not assimilate with, or be consistently united in, the building of a strong and substantial edifice, harmonious, beautiful and regular in all its proportions. As I have believed that *individual exertion* has done more to elevate the profession, than any societies which have hitherto been formed, as they have been conducted, so do I believe, that if *such men*, men of genius, of intellect, of high and exalted principle and purpose, were combined together in one united body, such an association, by its well directed efforts, might do much for the promotion of public and professional good, that individual exertion, isolated and alone, would fail to accomplish.

But the great difficulty has been, that such mortals were, "like angels' visits, few and far between," and could not be collected together for practical purposes. Hence the consequence. Societies have been formed, filled up with men of dissimilar feelings, tastes, sentiments, inclinations, differing greatly in mental as well as professional principles and attainments, and totally unfit to unite in the accomplishment of any object tending to the promotion of a legitimate system of correct practice.

In order to ensure the permanent existence and success of a society, the subject should engage the attention and secure the interest of every individual of known influence and character in the profession, within its precincts, (to the exclusion of all who are not qualified, in every respect, to assist in the work,) and with these it must be a common cause, or effort will be useless. The more exclusive such an association—the more fixed and peremptory in its restrictions—the more exacting in its requisitions, as to the attainments and qualifications of its members—the greater will be the desire and the ambition of all good men and true, to fit and prepare themselves to unite with it—and thus, and only thus, will it be certain to accomplish the great and praiseworthy object of its organization.

It is unnecessary to enter largely into the character of other associations to prove this. Most members of the profession are acquainted with their history.

A short notice of the American Society of Dental Surgeons—convened in the city of New York in the year 1840—commenced under favorable auspices, and with a degree of enthusiasm on the part of the few individuals who were its founders—but which was proved to have been built upon an insecure basis—may serve as one example, to illustrate the importance of laying the foundation stone sure and steadfast. A feeling of congratulation, of honorable pride, that we had assembled for the noble purpose of giving character to the profession—an object worthy the high ambition of its votaries—kindled in our hearts a generous, a charitable, but mistaken feeling of philanthropy, which induced us to extend the right hand of fellowship to *all* whom we thought had claims to be considered *reputable members* of the profession, that they might also assist in this great work.

This was the first great error, and the most fatal; it was the cause of all that strife, bickering and contention which marked its career:

> "A change came o'er the spirit of our dreams,
> That which was bright grew sad."

I, for one, had entered reluctantly into that association, in the beginning, because I doubted its success. I expressed my unwillingness to join it, and stated my fears that too much latitude would be given in the admission of members. There were others who had like doubts and misgivings. Its able and venerable first president, the lamented Dr. Hayden, who was the prime mover and founder of the institution, in the last conversation which I remember to have had with him, remarked, "that he had been disappointed in his fondest hopes, his long cherished aspirations—that the society was founded upon principles which he did not approve, that there had not been sufficient circumspection, and we had advanced too fast and too far, in much too short a time."

The first meeting, it will be remembered by members here present, was organized while he was so unwell as not to be able to attend. He, however, acquiesced in the proceedings, although he disapproved them, and to the end of his life continued to manifest a deep interest in its welfare, as he was accustomed to do in every undertaking in which he engaged.

"No more that venerable form appears,
But memory brings it from the turf-clad ground;
We seem to see again that lofty brow,
Calm and yet firmly fixed; that steadfast eye
Yet beaming mildly; and that head of snow
With all its worth and reverent majesty."

Let us, for the good of others, consider a little farther the errors of judgment in the formation of that society, and the consequences attendant upon the attempt to assimilate or amalgamate discordant elements—substances having no affinity for each other.

At a meeting of the American Society, held in this city, in the year 1841, not being able to be present, I addressed a communication to them, in which I proposed this question for discussion: "Are there cases in which it is essentially advisable and important that teeth should be filled, in which gold foil cannot be used, and other articles be substituted, so as to preserve the teeth a sufficient time to compensate for the operation?" I little thought then, gentlemen, what a protracted discussion that question would lead to. I little dreamed that I had inadvertently thrown a firebrand, which would ultimately cause such an extensive conflagration. But what was the immediate effect? What action did the society take upon this question at that time?

A committee, consisting of Drs. E. Parmly, E. Baker, S. Brown, C. A. Harris and J. Parmly, were appointed. This committee reported, "That the use of lithodeon, mineral paste, and all other substances of which *mercury* is an ingredient, were injurious to the teeth; and that there was no tooth in which caries could be arrested, and the organ rendered serviceable by being filled, in which gold could not be employed."

How was this report received? The report was *unanimously* adopted by the society. What effect did that have? Dit it lessen the spread of the evil? Has it counteracted in any degree the use of the article? Did it even have the power to prevent its own members from acting in direct violation of this, its own expressed judgment? No! the evil rather increased than diminished, and at every succeeding annual meeting, (except the last,) it was this vexed and agitated question, and this only, that was discussed, so that the true objects of the society were made null and void.

It was in view of this state of things, that when invited, in 1844, to deliver the annual address, I brought before the society a consideration of its constitution and its objects, as set forth in article 1st of its preamble.

The address which I had the honor to deliver on that occasion, Mr. Provost, as you well know, I declined furnishing for publication, for the reason that a great portion of it related to society matters only, and was rather intended for *secret session.* Not, sir, that I had any compunction for what I then uttered, and to prove that I am willing to receive my portion now, of that censure which some individuals think the American Society have incurred; to show what I then thought and still think, respecting an indiscriminate association of the profession, and the injury therefrom to society, I will extract a few sentences from that address, as follows:

"It is my purpose briefly to inquire how far this society has acted in accordance with its professed principles, with how much care the line of distinction has been drawn, how much it has accomplished, and how it is likely to succeed in bringing to a successful issue what remains to be done?

"Do gentlemen who are admitted into this association, think they have the right to act independently of its constitution, its laws, the expressed will of a majority in regard to what is, and what is not; what does, and what does not, constitute malpractice? There are such, there are those whom I have heard assert, that this society has not the right to control individual members. I trust and hope, sir, the society will use the power they possess, and will govern all who have signed the constitution, or cast them out. I do not cherish the belief that this society has done, or can do, any thing, by way of improvement, so long as it has upon its roll, the names of those, who have no fair claim to be considered as belonging to the profession; not educated in it, in any sense. This society has in it many members of the profession, for whom I have great respect, and with whom I am proud to be associated. Nor is it necessary that any member shall have attained great reputation or rank, to command my respect. It matters not what a man's occupation may have been previous to his turning his attention to this profession, so that he makes himself master of it. I ask not what he was, but what he is. Show me his works and I will tell you whether I can respect him. A highly esteemed correspondent writing to me upon this subject, used the following language: "Though I respect every man who follows a useful calling in an honest way, and am ready to take him by the hand, as a fellow laborer in the business of life, still, I would not, if I belonged to the honorable fraternity of blacksmiths, and wished to form a society, for the improvement or elevation of the trade, associate with miners, colliers, or bellows makers, with a view to advance my object, because their labors, had some remote bearing on the art."

To return from this digression. The present members of the American Society, had a hard and protracted struggle to obtain the ascendancy, and a painful duty to perform, to retain their power. I believe they have acted conscientiously, and that the society occupies a higher position now, though diminished in numbers, than it has ever done; and that it may be instrumental for the future, in accomplishing the design of its founders.

There was no alternative, the crisis had arrived, the majority must either have succumbed, and left the society in the hands of those, whom they considered advocates of a system of malpractice, giving them all the *eclat* of a victory, with the power to use the society for the advancement of their own ends; or retain the ascendancy, by pursuing the only course left them; (*arbitrary* it was deemed by the minority,) but the constitution authorized it, and an imperative sense of duty demanded it.

Of the Western Society, in the State of Ohio, I know but little. Judging from its publication, it bids fair to take a high stand; and if sufficiently exacting in its restrictions, and cautious in making merit and attainment indispensably requisite as qualifications for membership, it must exert a good influence upon the profession and the community in that section.

It has among its members, gentlemen of sound sense, and mature judgment. Its journal is well conducted, and I hope sincerely it will prove of great public utility.

I cannot, conscientiously, omit to speak as plainly, respecting the New York State Society, as I have of that of which I am a member. I should not be fulfilling my duty on this occasion of the formation of a new society, if I did not freely and explicitly inform you of every prominent corrigible defect, which may have been discovered in other societies. So long as we have no fixed laws, and constitutional barriers, for the security of our professional liberties and immunities, it is the province of every lover of truth, to stand up in their defence, and may "my tongue cleave to the roof of my mouth, and my right hand forget its cunning," if I remain inactive, when I see any course taken detrimental to my profession, and injurious to the community. I shall not "bend the pregnant hinges of the knee that thrift may follow fawning" to any individual or association.

The Society of Dental Surgeons of the State of New York, has been in existence nearly two years. Its *preamble* defines the object of its formation in these words:

"In order to create a more perfect union for professional improvement, by a free communication of facts and interchange of opinions—*and to promote the honor, character, and interests of the dental profession*, the undersigned dental surgeons, of the state of New York, have formed themselves into a society," &c.

In article 2nd, sections 3d and 4th of the constitution, we find some evidence of the *principles* of association, by which this desirable end was to be attained.

"Section 3. All persons in the practice of dental surgery, *at the time of the formation* of this society, may *become members on application;* provided, always, that they are persons of good moral character; that three-quarters of the members present vote for their admission; that they comply with the provisions of the constitution; *unless objections are made,* in which case, all such persons shall be referred to the executive committee, and if, after examination, they report favorably, they shall be elected as above described.

"Sec. 4. All persons who shall become practicing dentists *subsequent to the formation of this society,* who may apply for membership, *shall not be admitted unless upon examination,* found to possess competent knowledge of the *theory,* and have been in the study and practice of dental surgery at least two years."

Not one word is said in the whole constitution, respecting the importance or necessity of an examination, as to the *practical* skill or ability of any applicant. As if a knowledge of *theory* alone were the only indispensable requisite, by which to test a candidate's qualifications.

I am not actuated by competition, jealousy, envy, or malignity in what I shall say, respecting this society. For some of its members, I have always entertained a high respect, but I am opposed to it, (as I should be to any other in that state or out of it, conducted upon similar principles,) upon the broad consideration, that it is an imposition upon any community, as well as an injury to other associations, colleges, and the profession at large, for the reasons which I shall advance in the few following remarks.

In the *American Society,* the chief error, as I have before said, was too great an extension of the privilege of membership at the very outset, in the first convention.—What policy did its projectors adopt? What was the plan pursued? *Each person*, then present, was permitted to name such other gentlemen, as he considered would be a desirable acquisition, and they were enrolled, leaving it optional with them, to continue their connection afterwards. Fatal mistake! and yet with this example before them, and as if sufficient latitude had not been given, the originators of the *New York State Society*, (some of whom were *members* of the *American Society* at that very time, and had experienced all the difficulties, consequent upon that very error, up to the day of their separation from it,) issued notices throughout the state, inviting *all dentists* to assemble in convention, and after securing the passage of *article* 2d, *sections* 3d and 4th, which have been quoted, any one present, if no other objected, had the right to *sign the constitution*, and, *ipso facto*, he became a member.

Nay, more, in their enthusiastic zeal, and noble purpose, of advancing *the means of elevating* the profession, an attempt was made to pass an act, by which the society should confer a *diploma* upon each of its members. Was there no remonstrance against this assumption of power, this high-handed act of usurpation? Were there not men enough of sense, of discernment, even in an *amalgamation* meeting of this character, (however strongly they might favor the formation of the society,) to oppose such a flagrant violation of right? Thank heaven there were. In this enlightened age, we might well blush for the profession, if there had not been. A few individuals having heard that such things were to be advocated, and believing that if adopted, they would prove eminently detrimental to the standing and character of the profession in the city, and state, attended the meeting, not with the intention of opposing the formation of the society, but for the express purpose of remonstrating against these acts. Conferring honors and titles upon individuals in no way entitled to them! without any preparatory examination, (and in no way improved by a merely nominal association, with a few gentlemen of character and standing,) thus enabling them, with the advantages which a *diploma* confers, to secure for themselves a greater share of public confidence and patronage, without being any better qualified, or more worthy to receive it!

The minority had a right to remonstrate, and by earnest appeals to the better sense of the meeting, they succeeded in securing the defeat of *that act*. For this, and for some remarks which were made, respecting the injury which must accrue to the profession at large, by uniting in the bonds of association and fellowship, with respectable members of it, the *uneducated*, and it might be the *unprincipled*, we were stigmatized as *revilers* of the profession, and *disturbers* of the meeting. God help me! I for one could not see, nor can I yet, how a few educated individuals, even if in all respects qualified, theoretically and practically, to give instruction, are so to *educate all* belonging to the association throughout the state, by annual, monthly, or weekly meetings, if they could be convened so often, as to entitle their members to a *diploma*. I ask you, gentlemen graduates of this institution, how many years would it have taken to have acquired the knowledge, which you possess, under such a system of instruction? How much more difficult it is, too, to instruct those, who have settled down in a particular system, and mode of practice. There are many ignorant pretenders who have a most exalted opinion of their own merits; in their self-sufficiency they will allow no superior; possessing an overwhelming sense of their own importance, they cannot be convinced that there is any thing, "between heaven and earth, that they have not dreampt of, in their philosophy." It would be a hard task in a school like this to unlearn such, and teach them anew the first rudiments and principles of correct practice, and however much they might desire a *diploma*, they would consider it too great a sacrifice of their time and talents, to seek it here.

Mr. Provost, you were present, and no doubt recollect, when I was called to order in that meeting, and the gag-law enforced, *for too free an expression of opinion;* it was said by a distinguished member, in extenuation, that "he was acting upon *democratic principles*, and would not longer hear the *profession abused*."

I felt some slight twingings of conscience, for if assisting to form an association in which, attainments and qualifications for membership were a mere secondary consideration, was to be entitled, acting upon *democratic principles*, I too had once been a democrat. But this was many years previous, and if this is *democracy* in a professional point of view, I hope there are very few democrats at the present time, members of the *American Society of Dental Surgeons*.

With those members of the New York State Society to whom I am alluding, I am on terms of friendly intimacy, and I have no disposition to make a breach between us, nor do I believe I shall be proscribed for opinion's sake; but were my own brother to propagate such sentiments, I should dissent with him in opinion, and feel that I had a right to freedom of speech, and would oppose him on all occasions, in a fair and honorable, yet amicable, disposition.

Believing such a society to be a public injury, I say to those of you who are about establishing yourselves in different sections of these United States, if you are ever called upon to assist in forming *local societies, weigh well these things.* Draw your own conclusions. I may be wrong. Judge ye for yourselves.

I had intended, feeling it to be a public duty which some one ought to perform, long since to have noticed this society, but so favorable an occasion as the present has not been presented. All societies and associations should know each other, and the principles, laws and regulations, by which each is governed, and having often been asked the question if I was not a member of the New York Society? And why not? I am constrained to publish and present my objections to it. Look on that *picture*, gentlemen, it is a wood engraving, which has been circulated in the city of New York, as one of the *ways and means*, of securing public patronage. It is presented to the world, ostensibly, by a member of that society, apparently countenanced by a body of respectable gentlemen, associated for the *elevation* of the profession.

Truly, Mr. President,

"The earth hath bubbles,
As the water hath; and such are these."

Let it not be thought, that in exercising the duties of the office to which you have invited me, I am arrogating the authority to judge of men or measures, in thus earnestly and plainly expressing myself.

I am not unconscious that benevolence, charity, mercy, are the most beautiful attributes of man's nature, and if I have said aught that may appear to militate against these, in considering the character of those who claim to be followers of the same calling as myself, it is because my duty to society is paramount. "It is not that I love Cæsar less, but that I love Rome more." I am here to advocate the right and importance of establishing secure professional safeguards, defences, barriers, against the multitude of unprincipled and reckless practitioners, who are imposing themselves upon the community as members of the profession; not to advocate measures for their protection and encouragement.

We may pity those whom we are compelled to denounce as unworthy of public confidence. We may feel that

> "——the veriest wretch on earth,
> Doth cherish, in some corner of his heart,
> Some thought that makes that heart a sanctuary,
> For pilgrim dreams in midnight hour to visit,
> And weep and worship there."

We may have a free flowing of the milk of human kindness, which softens life's asperities, and be actuated in disposition and conduct, by this beautiful sentiment, as a light to guide us in our earthly pilgrimage:

> "The quality of mercy is not strained,
> It droppeth as the gentle rain from heaven,
> Upon the place beneath; it is twice blessed,
> It blesseth him that gives, and him that takes."

We may feel all this in our inmost heart, yet justice demands that we should probe the depths of degradation into which so many are fallen; a sense of duty impels us to point out the way in which prejudice and ignorance have done their work of wrong upon the innocent and injured.

We cannot sacrifice realities to the morbid chimeras of prurient philanthropy. That wisdom and knowledge, derived from a deep and earnest contemplation of the subject, must dictate the imperative necessity of acting as *public monitors*—of sacrificing all feelings of individual love and affection, no matter how near or dear, upon the altar of professional duty.

There is a light to guide men of clear heads and honest hearts, in every case of emergency: it is the light of truth. There is an inward monitor to direct: the still small voice—the voice of conscience. Let us then do our duty, regardful only of the interests, safety and protection of the millions who are so much dependent upon our professional good offices—regardless of the voice of vituperation—undismayed, though villified and denounced—fixed and unalterable in a determination to do effective service in the cause of humanity.

Having now, *Mr. President and Gentlemen of the associated Alumni of the Baltimore College*, impressed you with my views of what a society should, as well as what it should not be, it is for you to pursue the readiest measures which shall suggest themselves, to ensure the success of your society, and its continued progress and advancement. I can only express, in conclusion, the hope that you will continue unceasingly your endeavors to render it a powerful agent for the promotion of good, leaving no means untried, and no efforts spared, to further this object. What influence it may be destined to exert over the future, we cannot predict; but if you judge rightly of the past, if you gather wisdom from experience, and improve by it, you have everything to hope for.

*Gentlemen Graduates* of the Baltimore College, I cannot permit this opportunity to pass without saying a few words to you, expressive of the deep interest we take in your future welfare and success. You will have the pleasure of hearing, this evening, an instructive and eloquent address, from a gentleman whose professional lore and experience will enable him to shed light upon your pathway to professional attainment; and I trust I shall in no degree trespass upon his official province, in presenting a few plain practical remarks for your consideration.

A gentleman called upon me a short time since, from the far south: in the course of conversation, he remarked, in substance, "that he had been for many years a dental practitioner; had received, before starting upon his career, a good education; had read medicine and surgery for two years, but had not graduated at any college. He said he had deep reason to regret this omission;" and when asked in what particular respect, he replied, "that since that time dental colleges and societies had sprung into existence; that he was totally ignorant of their origin, principles or measures, unacquainted with their governments, presumed them to be desirable and useful objects, but that they had sent so many out into the world, bearing their broad seals, and he himself being entirely out of their pale, he began to think he was only a quack after all, when he had believed for many years that he was a good and efficient operator." I inquired if he thought that *titles* and *distinctions* conferred upon individuals secured them any peculiar privileges, or advantaged them in gaining the confidence and patronage of a community. His answer was to this effect: "wherever the graduates of the Baltimore College have gone out into the south, they have taken away the business of old practitioners. *Their diplomas* are a passport everywhere, and the people believe that a graduate from that college knows more than any one else." Now, gentlemen, if this be so, what does it prove? Why, reaction! reaction in public sentiment, the gradual yet forcible reaction which is progressing in the popular mind. It proves that the community are willing to repose confidence in those only whom they securely feel may be trusted. It presages the downfall of charlatanism and the triumph of true science. It assures us that the day for newspaper puffing paragraphs, and the advertising circulars of itinerant mountebanks, the unblushing effrontery of self-taught performers of an art, upon which depends so much of human comfort, health and happiness, is rapidly drawing to a close. But if these things are so, gentlemen, to all graduates of this college, a weighty responsibility attaches. May you feel it, in all its importance. I beseech you to remember that you are not alone laboring in your vocation for increase of wealth—but also for increase in knowledge and usefulness—for fame as well as riches. Riches are but the means of life, not its object. "A good name is rather to be chosen than great riches." Remember, each of you, that the success of his fellows of this institution, and all connected with it, in furthering the great object of professional improvement, for the promotion of science, and the rights of humanity, is in your hands.

There is one word, which I desire to impress upon your minds, that it may have an abiding influence, and a few remarks connected therewith, will conclude this address.

*Doubt*—I repeat it, *doubt;* not your ability to do well; not, that the knowledge you have here acquired is your best capital; not, that it is possible for you ultimately to reach the highest attainable point of professional excellence. The *divine law*, which regulates and governs all space, all matter and all mind, happily fixes no limit to man's energies, nor interposes any barrier to his progress. Yet again I say, *doubt;* doubt, to some extent, the *permanency* of your operations. Do not feel sure, (I speak particularly in reference to the operation of *filling teeth,)* do not feel certain that any operation is to be permanent. Do not say to your patient, "*You may be assured that filling will last you ten, twenty or fifty years.*" Though you may be as sure of its durability as man can be of anything here below, do not say it. Doubt even when you think there is the least occasion for doubting. You may wonder why I lay so great stress upon this: the secret of your professional improvement and success depends in no small measure upon it. Believe me, there is no more fatal error that you can fall into, in your early practice, than an absolute confidence in your own infallibility. Let the thought cross your mind, and ask yourselves the question, have I done all I might have done, in that last operation, to make it permanent? Always have the impression indelibly fixed in your mind, that there is something more to be acquired, some little improvement continually to be made.

Your standard should be planted high above you, where you can behold it. Upon its folds, emblazoned the word—*Excelsior*. Let your aim be to climb the mountain, but never expect to reach the highest pinnacle. Perfection is not for mortals. Absolute perfection is not in human nature. Strive with your contemporaries in all honorable endeavors to see who shall approach nearest to it. Do this, and you will attain the extent of your earthly excellence. See that the confidence reposed in you is not betrayed. Be faithful to your trust, and it will redound to the honor of this institution, and all who go out from it, hereafter, will receive their reward, and reap the benefit of your well doing. Let love to your fellow-men, and the desire to do good, be the main spring of all your movements.

There is a beautiful fable of oriental origin, that speaks to the hearts of all, in the *moral* which it inculcates:

"Abon Ben Adhem, (may his tribe increase,)
Awoke one night from a deep dream of peace,
And saw, within the moonlight in his room,
Making it rich, and like a lily in bloom,
An angel, writing in a book of gold.
Exceeding peace had made Ben Adhem bold,
And to the presence in the room he said,
'What writest thou?' The vision raised his head,
And in a voice made all of sweet accord,
Answered, 'The names of those who love the Lord!'
'And is mine one?' said Ben Adhem. 'Nay, not so,'
Replied the angel. Abon spoke more low,
But cheerly still, 'I pray thee, then,
Write me as one who loves his fellow-men.'
The angel rose and vanished. The next night
He came again, with a great wakening light,
And showed the names whom love of God had blest;
And lo! Ben Adhem's name led all the rest."

